# Reliability Analysis of Observation-Based Exposure Assessment Tools for the Upper Extremities: A Systematic Review

**DOI:** 10.3390/ijerph191710595

**Published:** 2022-08-25

**Authors:** Preston Riley Graben, Mark C. Schall, Sean Gallagher, Richard Sesek, Yadrianna Acosta-Sojo

**Affiliations:** 3323 Shelby Center for Engineering Technology, Department of Industrial and Systems Engineering, Auburn University, Auburn, AL 36849-5346, USA

**Keywords:** musculoskeletal disorders, occupational safety and health, physical health, prevention and protection, risk perception and management, ergonomics, risk assessment, fatigue failure

## Abstract

(1) Background: The objectives of this systematic review were to (i) summarize the results of studies evaluating the reliability of observational ergonomics exposure assessment tools addressing exposure to physical risk factors associated with upper extremity musculoskeletal disorders (MSDs), and (ii) identify best practices for assessing the reliability of new observational exposure assessment tools. (2) Methods: A broad search was conducted in March 2020 of four academic databases: PubMed, Science Direct, Ergonomic Abstracts, and Web of Science. Articles were systematically excluded by removing redundant articles, examining titles and abstracts, assessing relevance to physical ergonomics and the upper extremities, and article type. (3) Results: Eleven articles were included in the review. The results indicated no singular best practice; instead, there were multiple methodological approaches researchers chose to use. Some of the significant variations in methodologies include the selection of reliability coefficients, rater and participant selection, and direct vs. digital observation. (4) Conclusion: The findings serve as a resource summarizing the reliability of existing observational risk assessment tools and identify common methods for assessing the reliability of new observational risk assessment tools. Limitations of this review include the number of databases searched, the removal of truncation symbols, and the selection of keywords used for the initial search.

## 1. Introduction

Work-related musculoskeletal disorders (MSDs) of the upper extremities, the body regions comprised of the shoulders, arms, wrists, and hands, are prevalent and burdensome conditions [1,2]. MSDs can be detrimental to one’s quality of life at work and home. According to the United States Bureau of Labor Statistics, an average of 271,126 MSDs involving days away from work were reported annually from 2016 to 2020. Of those MSDs reported in 2020, 77,800 (31.42%) were to the upper extremities [3]. These cases required a median of 21 days away from work. Carpal tunnel syndrome (CTS), one of the most burdensome MSDs of the upper extremity [4,5], has been estimated to affect 0.6–2.1% of men and 3.0–5.8% of women in the general population [6,7,8]. The prevalence of CTS in a pooled multicenter cohort of 4321 primarily industrial workers was higher, at 7.8% (95% CI of 7.1–8.6%) [9].

The direct costs of upper extremity MSDs are substantial. Webster and Snook [10] reported that the average compensable cost of an upper extremity MSD was USD 8070 in 1989, which translates to approximately USD 17,430 in 2021 when adjusted for inflation using the Bureau of Labor Statistics Consumer Product Inflation (CPI) calculator [11]. Surgical release treatments for CTS are carried out approximately 450,000 times annually in the U.S., costing approximately USD two billion (not including the much higher estimate of associated indirect costs such as job change, retraining, and lost work time) [12]. Alarmingly, estimates suggest that only 6–8% of upper extremity MSDs are ever reported [13], suggesting that the problem may be more significant than is apparent.

The pathology of upper extremity MSDs has been linked to occupational exposure to physical risk factors, including vibration, contact stress, lack of recovery time, excessive force, repetition, and non-neutral postures [14,15]. Identifying and quantifying exposure to physical risk factors in industry is imperative to prioritize operational improvements. Ergonomists have developed observation-based exposure assessment tools to assess work tasks to identify and mitigate some physical risks associated with employees’ jobs [16,17]. Common examples include the American Conference of Governmental Industrial Hygienists (ACGIH) threshold limit value for hand activity (TLV for H.A.) [18,19,20], the Strain Index [21], Rapid Entire Body Assessment (REBA) [22], and Rapid Upper Limb Assessment (RULA) [23]. These tools combine aspects of exposure to provide an overall assessment score of a job that can measure how “risky” that job is or how likely it is that an MSD will result.

One of the weaknesses of several ergonomic tools is their subjective nature. Reliability measures the capability to replicate measurements of the same object of study [24,25,26,27]. Typically, reliability is broken into two components: inter-rater and intra-rater reliability [28]. Inter-rater reliability refers to the ability of two or more raters to produce the same results when using the tool [24,27,28]. Intra-rater reliability (also known as test–retest repeatability) is the ability of a single rater to replicate past measurements using the same tool for the same unchanged job or task [24,27,28]. These measures are critical for job analysis, since similar results are desirable regardless of the rater [29].

Ultimately, because no ergonomics exposure assessment tools are identical, the methods used to evaluate their reliability (statistics and study design) will most likely differ. The difference in reliability metrics can make it difficult to compare and contrast the reliability of different tools. Furthermore, the reliability of some tools may have been more comprehensively studied. Consequently, selecting an observational exposure assessment tool for assessing exposure to physical risk factors associated with MSDs of the upper extremity can be challenging for many occupational safety and health professionals.

The objectives of this systematic review were to (i) summarize the results of available reliability studies of observation-based ergonomics exposure assessment tools used for assessing exposure to physical risk factors associated with upper extremity MSDs, and (ii) identify best practices for assessing the reliability of new observational exposure assessment tools. The results may be helpful for ergonomists interested in evaluating and selecting available observation-based exposure assessment tools for their work and identifying methods investigators may apply to assess the reliability of new observation-based risk assessment tools.

## 2. Methods

### 2.1. Article Database Selection and Search Strings

**Figure 1:**
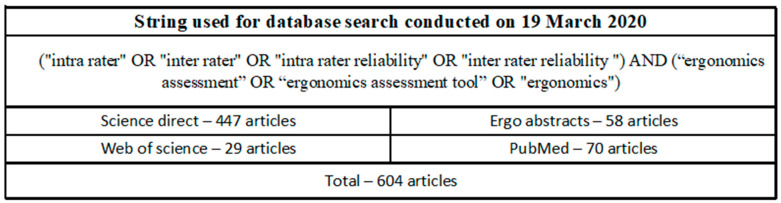
Search string used in the review.

The review team was comprised of two industrial engineering doctoral students and three tenured engineering professors professionally certified by the Board of Certification in Professional Ergonomics. Four academic databases were searched for articles, including Web of Science, Ergonomic Abstracts, PubMed, and Science Direct. The databases were selected with consideration to the field of research and are commonly used by researchers in ergonomics. To ensure a comprehensive review, the review team selected keywords for the search string used in the review to encapsulate ergonomic exposure assessment tools and to categorize those tools based on body segment (Figure 1). The initial database search was completed on 19 March 2020.

### 2.2. Article Review Process

**Figure 2:**
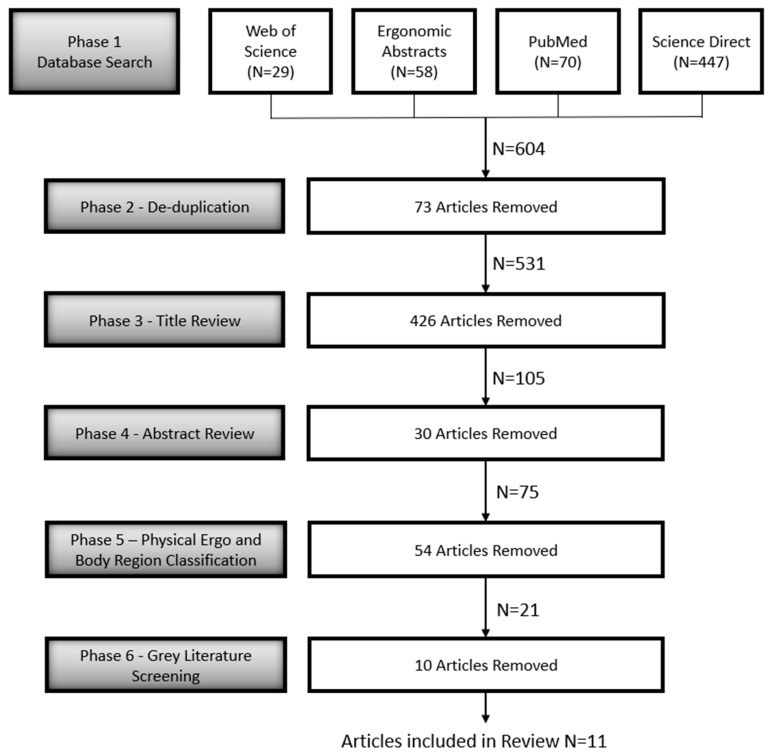
The article selection process.

The article review process is illustrated in Figure 2.

After the initial database search was performed, duplicate articles were removed. The two student reviewers then independently reviewed articles for consideration based on the relevance of the article’s title. Only articles deemed non-relevant by both student reviewers were eliminated from further consideration. Disagreements were settled through discussion by the entire review team until a consensus was reached. The same method was used for the review of abstracts.

The 75 remaining articles were first separated based on whether they focused on physical or cognitive aspects of work. Next, articles unrelated to the upper extremity (including the shoulders, arms, wrist, and hands) were removed. Only one additional article [30], which used direct measurements, surface-electromyography (SEMG) and a hand-held dynamometer, was removed from the review, since the review focuses on the subjective, observational aspects of ergonomic assessments and not direct measurement. There were no articles that implemented self-reported data. Finally, the grey literature (systematic reviews and conference proceedings) and non-English articles were removed.

Three additional studies were removed from the review as they implemented software to gather the exposure data [31,32,33]. The study by Abobakr et al. [31] used software to gather the inputs necessary to calculate a Rapid Upper Limb Assessment (RULA) score. Levanon [32] collected biomechanical data via an infrared motion capture system to evaluate tasks. Manghisi et al. [33] used the Microsoft Connect System to collect data to calculate a RULA score. That score was then compared to an optical motion capture system and a RULA expert rater to assess reliability. These methodologies bypass an observer evaluating a task and entering the data by hand, which is the typical observational approach. Since a human did not collect the data, it removes an element of human error. Therefore, its reliability should not be compared against similar assessments conducted by humans, leading to their removal from the review.

## 3. Results

Eleven articles were included in the final review (Table 1). Differences in the reviewed studies include sample size (number of participants), number and type of raters, the use of video files (Table 2), and the selection of reliability coefficient(s) (Table 3). A complete summary of the reliability results can be found in (Table 4). It is worth noting that the Strain Index was one of the most heavily researched tools and consistently had high reliability in our sample, especially when used in a team environment. In this review, “participants” are the workers selected to perform tasks for the “raters” to observe and analyze using the assessment tools.

### 3.1. Observational Measurement Technique: Field Observations vs. Video Recordings

All of the articles included in the review used observational techniques. Three studies were conducted in the field, meaning the rater(s) observed the process and the worker in real-time. The remainder (*n* = 8) utilized observational techniques via video recording. A participant would be recorded completing a task or series of tasks in these studies, usually from more than one vantage point. Then, the raters would view these videos and use the observation-based assessment tools to analyze the task(s). Most of the studies in this review used video recordings, which may indicate that this is the preference in industry. However, it is unclear if one methodology should be the accepted practice.

### 3.2. Sample Size and Raters

Sample size was listed as a limitation in many of the articles reviewed. All articles reviewed used a sample size in the range of 5–15 raters. A limited number of raters was typically due to resource constraints. A limited number of raters can also result from inclusion criteria, such as the study conducted by Rhen and Forsman [34], who selected only raters that were ergonomists with exposure assessment experience. A recommendation from the literature suggests that at least 30 raters should be included to assess reliability based on the desired confidence interval [35].

The selection of raters was also a source of variation within the articles reviewed. Many studies included students [16,26,29,36,37,38,39,40] and university faculty [16,36,40] as their raters. Most raters had prior knowledge and experience in physiotherapy and/or ergonomics. Some raters were professionally certified as ergonomists and/or licensed physiotherapists [16,29,34,38,41]. Many raters were selected for their experience as practicing ergonomists [26,29]. All raters received training on the tool relevant to the study, ranging from a few hours to sometimes many months, and had some previous knowledge of ergonomics.

### 3.3. Reliability Coefficients

Many statistical methods may be used to evaluate reliability [27,28,42]. Since various statistical methodologies are used to measure reliability, it is vital to understand how they differ and their appropriateness for various reliability studies [29]. This section will briefly discuss the reliability statistics used in the reviewed articles. Information regarding the reliability coefficients can be found in Table 3.

Cohen’s kappa is one of the most popular coefficients used in reliability studies, but it can only be used to analyze categorical data [27,28]. Four of the eleven reviewed articles used a version of Cohen’s kappa as one of the reliability coefficients. In kappa statistics, the measure of agreement between raters or within a single rater is compared to the agreement expected to occur by chance alone [43,44]. Using Cohen’s unweighted kappa means that all ratings that are not identical are “punished” equally. In other words, the degree to which the raters’ ratings differ is not considered [34]. To overcome some of the weaknesses of Cohen’s kappa, one could use weighted kappa since it allows responses to be weighted as a function of the level of disagreement as determined by the researcher [27]. The weighted kappa is calculated with respect to a weighting system that discriminates against major and minor differences in ratings [34,45], and what constitutes a major or minor difference is determined by each study’s authors.

**Table 1:** Title, industry of interest, and stated objectives of the articles included in the review ^a^.

Author (Year)	Title	Industry	Stated Objective
Neumann et al. (1998) [46]	A participative field study of the inter-rater reliability of a risk factor assessment checklist used by manufacturing plant personnel	Foam manufacturing.	The purpose of this study was to evaluate the inter-rater reliability of the Manufacturing Operation Risk Factor Checklist (MORF) in a realistic field implementation.
Dockrell et al. (2012) [38]	An investigation of the reliability of Rapid Upper Limb Assessment (RULA) as a method of assessment of children’s computing posture	Elementary school.	The objectives were to (1) to establish the inter-rater reliability of RULA in children (2) to establish intra-rater reliability of RULA in children (3) to investigate the association, if any, between child’s age and reliability of RULA.
Rhen and Forsman (2020) [34]	Inter- and intra-rater reliability of the OCRA checklist method in video-recorded manual work tasks	Grocery and cashier work, meat deboning and netting, engine assembly, lavatory and stair cleaning, post-sorting, and hairdressing.	The objectives were to, with respect to risk factors and calculated risk levels, study the consistency of (1) assessments performed by different ergonomists (inter-rater reliability) and (2) repeated assessments performed by each of the ergonomists (intra-rater reliability) of the Occupational Repetitive Actions (OCRA) checklist.
Paulsen et al. (2014) [40]	Inter-rater reliability of cyclic and non-cyclic task assessment using the hand activity level in appliance manufacturing	House appliance manufacturing.	The purpose of this study was to compare the inter-rater reliability of the HAL assessments used to estimate worker exposure to repetitive hand extensions during cyclic and non-cyclic task performance in the appliance manufacturing industry.
Stevens et al. (2004) [29]	Inter-rater reliability of the Strain Index	Videos were selected from an archive to provide a full spectrum of rating categories for the task variables of the Strain Index.	The purpose of this study was to evaluate the inter-rater reliability of the Strain Index.
Dartt et al. (2009) [37]	Reliability of assessing upper limb postures among workers performing manufacturing tasks	Appliance manufacturing.	The purpose of this study was to determine the inter- and intra-rater reliability of assessing neck, shoulder, and wrist postures by using the Multimedia Video Task Analysis (MVTA)
Valentim et al. (2018) [41]	Reliability, Construct Validity, and Interpretability of the Brazilian version of the Rapid Upper Limb Assessment (RULA) and Strain Index (SI)	Textile industry, electronics industry, assembling line, tinsmith and sawmills, self-employed workers (hairdresser, dentist, beautician, woodworker, butcher, bricklayer, etc.).	The study aimed to cross-culturally adapt and test the measurement properties of the RULA and the Strain Index.
Stephens et al. (2006) [26]	Test–retest repeatability of the Strain Index	Manufacturing, meat/poultry, manual material handling.	The purpose pf this study was to investigate the test-retest repeatability of the Strain Index.
Paulsen et al. (2015) [16]	The inter-rater reliability of Strain Index and OCRA Checklist task assessments in cheese processing	Cheese manufacturing.	The purpose of this study was to characterize the inter-rater reliability of two physical exposure assessment methods of the upper extremity, the Strain Index, and OCRA checklist.
Hollak et al. (2014) [39]	Towards a comprehensive Functional Capacity Evaluation for hand function	More than 180 different occupations.	The purpose of this study was to develop a more efficient (shortened) protocol for hand function capacity evaluation and to test the agreement of the protocol compared to the original protocol.
Coenen et al. (2014) [36]	Validity and inter-observer reliability of subjective hand-arm vibration assessments	Laboratory.	Measuring hand-arm vibration objectively is often difficult and expensive, while often used information provided by manufacturers lack detail. Therefore, this study aimed to test a subjective hand-arm vibration assessment method for validity and inter-observer reliability.

^a^ The table may include direct quotes to maintain consistency with the original articles. Please seek the original articles for further information.

**Table 2:** Participant and Rater information for the articles included in the review ^a^.

Author (Year)	Number of Participants	Participant Demographics	Rater Training	Rater Characteristics
Neumann et al. (1998) [46]	8	N.A.	7–10 h of training on the use of the checklist.	Plant ergonomic committee members.
Dockrell et al. (2012) [38]	24	Children	Raters were given 45 min training sessions including a lecture and demonstration using PowerPoint on Rapid Upper Limb Assessment (RULA). It was followed by a practical session where they used the tool and compared and discussed their ratings.	Undergraduate physiotherapy students and experienced physiotherapists. Mean age of students = 22.2 years (range = 21–24. The mean age of therapists = 37.3 years (range = 31–45).
Rhen and Forsman (2020) [34]	One voluntary worker for each job filmed	N.A.	Raters were given a 25 min lecture and an Internet-based education on Occupational Repetitive Actions (OCRA), which included background, application, and a demonstration.	Licensed female physiotherapists with more than 4 years ergo experience as professional ergonomists
Paulsen et al. (2014) [40]	385 workers	Mean age = 42.3 years (SD = 10.6). Average experience = 14.7 years (SD = 11.4); 91.5% were white, 51.3% were males.	Each faculty member at the University was thoroughly trained in the use of the Hand Activity Level (HAL). They thereafter trained their graduate students.	Two university faculty members with extensive experience and nine graduated students trained by the faculty. Mean age = 29.8 years (SD = 8.6) and roughly 54.5% were female.
Stevens et al. (2004) [29]	The research team used video files and did not include any participant information.		All raters participated in a 1-day training course given in their respective geographic location. Lasting approximately 8 h, it included a description of the principles and procedures of the Strain Index, applied examples using video of real-world examples, along with feedback and discussion regarding the choice of the appropriate ratings.	Nine raters were practicing ergonomists and six raters were students studying for advanced ergonomic degrees.
Dartt et al. (2009) [37]	20	Mean age = 47.8 years (range = 34–62). Average experience = 19.7 years (range = 6–36; 50% were male.	Six months of software familiarity and two weeks of formal training sessions including (1) observing a professional (2) completing the same review as the professional (3) reviewing tasks and having the professional check their work afterwards (4) then completing analyses on their own.	Graduate students working in the Ergonomics Laboratory at Colorado State University.
Valentim et al. (2018) [41]	116 assumed workers for each job	N.A.	Each rater received additional training which consisted of explanations of the methods and theoretical/practical application of the assessment tools.	Each rater was experienced with three to five years of biomechanical exposure assessments.
Stephens et al. (2006) [26]	Assumed one worker for each job on the video file	N.A.	Each rater regardless of experience was given an 8 h tutorial on using Strain Index which included background on Strain Index principles, Strain Index applications, video file examples of jobs, demonstrations on how to apply ratings to video files, and an open discussion of example results.	Six graduate students (three masters and three PhD’s) and nine ergonomic practitioners. No Certified Professional Ergonomists (CPEs).
Paulsen et al. (2015) [16]	Assumed one worker for each job on the video file	N.A.	Training sessions included instruction on the procedures of each method, practice applying the methods to video segments of manufacturing tasks, and feedback from an experienced rater. Training sessions continued until trainees achieved competency. Competency for each method was reached when trainees consistently (80% of time) assigned exposure ratings that were similar (within 20%) to the most experienced rater.	Members from occupational health research groups including three university faculty and four graduate students. Two were CPEs.
Hollak et al. (2014) [39]	643 healthy working participants	402 mean and 241 women. Mean age = 41.6 (SD = 10.4).	Two-day Functional Capacity Evaluation training given by a licensed WorkWell trainer specifically for the purpose of this study.	Physical therapy students.
Coenen et al. (2014) [36]	2	Two males aged 37 and 56 with substantial knowledge and experience with power tools.	Each rater had substantial knowledge in human kinematics and ergonomic risk assessments but not regarding vibration; therefore, all received verbal and written instructions on the hand-arm vibration assessment.	Students and employees of Vrije Universiteit Amsterdam, Faculty of Human Movement Sciences and TNO Healthy Living. Mean age = 30.2 (SD = 12.1).

^a^ The table may include direct quotes to maintain consistency with the original articles. Please seek the original articles for further information.

**Table 3:** Observation information, reliability assessment(s), and interpretation for the articles included in the review ^a^.

Author (Year)	Number of Raters	Obs	What was Observed	Reliability Assessment	Interpretation
Neumann et al. (1998) [46]	7	56	Eight jobs.	Intraclass correlation coefficients (ICCs), similar index and comparable to the kappa coefficient, were calculated from 2 × 2 analysis of variance (ANOVA)	Poor-fair at ICC < 0.4, fair-good at 0.4 < ICC < 0.75, and excellent at ICC => 0.75
Dockrell et al. (2012) [38]	6	144	Twenty-four school children based on Shoukri et al. (2004) recommendation of 18–29.	ICC (2,1), ICC (3,1)	ICC < 0.50 = Poor, 0.05 < ICC < 0.75 = Moderate, ICC > 0.5 = Good
Rhen and Forsman (2020) [34]	11	220	Ten video recordings were analyzed twice by each rater.	Cohens linearly weighted kappa, ICC (2,1), Kendall’s coefficient of concordance KCC, percentage agreement	kappa < 0.00 = Poor, 0.00–0.20 = Slight, 0.21–0.40 = Fair, 0.41–0.60 = Moderate, 0.61–0.80 = Substantial, 0.81–1.00 = Almost Perfect, Percent agreement > 80% = acceptable, (ICC < 0.50, “poor”, 0.50–0.75 “moderate”, 0.75–0.9 “good”, and >0.90 “excellent” reliability)
Paulsen et al. (2014) [40]	11 working in pairs. Each person in each pair rated tasks individually, but each task was rated by one pair.	1716	385 workers doing 858 tasks	For each rater pair, reliability was measured between the scores using Pearson Product Moment Correlation Coefficient (Streiner and Norman, 2006) [27] and Two-sample Student’s *t*-test using Satterhwaite’s method for unequal variance was used to investigate cyclic vs. non-cyclic tasks.	Weighted Mean Correlation Coefficients—negligible: 0.00–0.25; fair to moderate: 0.25–0.50; moderate to good: 0.50–0.75; good to excellent: 0.75–1.0
Stevens et al. (2004) [29]	Fifteen raters and five teams.	1095	61 videos for specific task variables of the Strain Index and 12 videos for complete analysis (73 total).	ICC (2,1) using single measure and absolute agreement were used to analyze the data, task variable ratings, and Strain Index score. The Kuder and Richardson’s Equation 20 (KR-20) and percent agreement was used to analyze the dichotomized hazard score.	Poor-fair at ICC < 0.4, fair-good at 0.4 < ICC < 0.75, and excellent at ICC => 0.75. The authors did not indicate what other interpretations they used for the other reliability coefficients.
Dartt et al. (2009) [37]	2	80	20 jobs were analyzed twice by both raters	Generalizability theory paired with Pearson Product Moment Correlation Coefficients	Coefficients > 0.75 = good to excellent, 0.50 < Coefficients < 0.75 = fair to good, Coefficients < 0.50 = poor
Valentim et al. (2018) [41]	2	464	116 recorded tasks were analyzed twice by each rater	Kappa, ICC (2,1), percentage agreement, standard error for measurement, Cronbach alpha Coefficient, Spearman’s Rho.	Kappa (k < 0.00 = Poor, 0.00–0.20 = Slight, 0.21–0.40 = Fair, 0.41–0.60 = Moderate, 0.61–0.80 = Substantial, 0.81–1.00 = Almost Perfect), ICC (poor <0.40, moderate 0.40–0.75, strong 0.75–0.90, excellent >0.90), agreement (very good <5%, 5% < good = 10%, 10% < doubtful = 20%, negative >20%), Cronbach alpha (positive = 0.70 and 0.95, low < 0.70, redundant > 0.95), Spearman rho (weak = 0–0.30, moderate = 0.30–0.70, strong = 0.70–1.0)
Stephens et al. (2006) [26]	15 individual raters in 5 teams of 3	1854	73 job files (61 task variable and 12 Strain Index score files)	ICC (2,1) was used for most of the data while the tetrachoric correlation coefficient was used for the dichotomous hazard classification value	The authors of this study do not reference a single interpretation scale for either the ICC or the Tetrachoric Correlation Value.
Paulsen et al. (2015) [16]	3 university faculty and 4 graduate students for a total of 7 raters	448	21 cyclic U.E. tasks were to be analyzed; 11 were asymmetric and treated separately which increased the total tasks to 32	ICC(2,1)	ICC < 0.40 = poor reliability; 0.40 < ICC < 0.75 = moderate to good reliability; and ICC > 0.75 = excellent reliability
Hollak et al. (2014) [39]	1 of 15 physical therapy students	643	643	One-way random ICC(1,1) and Limits of Agreement (LoA)	0.91 < ICC < 1.0 (Excellent Agreement), 0.75 < ICC < 0.90 (High Agreement), 0.50 < ICC < 0.75 (Moderate Agreement) The LoA were assumed to be acceptable for clinical interpretation at 16%.
Coenen et al. (2014) [36]	16 in teams of 4	64	16 tasks	ICC, weighted Cohen’s kappa (k), and Percentage Agreement	For both ICCs and Ks >0.60 = good, 0.40–0.60 = agree moderately, <0.40 = limited agreement. To test for a learning effect, the percentage of agreement between subjective assessment and objective measurements in the first two tasks were compared to the last two tasks.

^a^ The table may include direct quotes to maintain consistency with the original articles. Please seek the original articles for further information.

**Table 4:** Reliability results for the articles included in the review ^a^.

Author (Year)	Reliability Results
Neumann et al. (1998) [46]	Reliability, as assessed using the intra-class correlation coefficient (ICC), was found to be poor for the upper limb, moderate for the torso and lower limb, and good for the assessment of manual material handling.
Dockrell et al. (2012) [38]	Rapid Upper Limb Assessment (RULA) demonstrated higher intra-rater reliability than inter-rater reliability, although both were moderate to good. RULA was more reliable when used for assessing older children (8–12 years) than with younger children (4–7 years). RULA may prove useful as part of an ergonomic assessment, but its level of reliability warrants caution for its sole use when assessing children, and in particular, younger children. Action Limit—Mean = (0.60), Standard Deviation (SD) = (0.20), Range = (0.59); Grand Score—Mean = (0.68), SD = (0.15), Range = (0.37); Arm Score—Mean = (0.62), SD = (0.25), Range = (0.58); Trunk and Leg Score—Mean = (0.75), SD = (0.13), Range = (0.32).
Rhen and Forsman (2020) [34]	For the five risk levels, the inter-rater overall percentage agreement was 39% and Cohen’s linearly weighted kappa was 0.43. For the six risk factors, the linearly weighted kappa values were between 0.25 (Posture) and 0.40 (Duration and Force). As expected, a higher (however just slightly higher) reliability was found within raters than between raters, with an overall percentage agreement of 45% and a linearly weighted kappa of 0.52. The linearly weighted kappa values of the risk factors ranged from 0.41 (Recovery) to 0.61 (Duration).
Paulsen et al. (2014) [40]	Results indicated that the Hand Activity Level (HAL) is a reliable exposure assessment method for cyclic ($\bar{r}$-bar_w_ = 0.69) and non-cyclic work tasks ($\bar{r}$-bar_w_ = 0.68). When the two reliability scores were compared using a two-sample Student’s *t*-test, no significant difference in reliability (*p* = 0.63) between these work task categories was found. This study demonstrated that the HAL may be a useful measure of exposure to repetitive exertions during cyclic and non-cyclic tasks.
Stevens et al. (2004) [29]	For task variables and estimated data, ICC (2,1) varied between 0.66–0.84 for individuals and 0.48–0.93 for teams. The Strain Index score had an ICC (2,1) of 0.43 and 0.64 for individuals and teams, respectively. For the most important variable, hazard classification, the Kuder and Richardson’s Equation 20 (KR-20) was 0.91 for the individuals and 0.89 for the teams.
Dartt et al. (2009) [37]	The results demonstrated good to excellent inter-rater reliability for neck and shoulder postures and fair to excellent inter-rater reliability for wrist postures. Intra-rater posture assessment demonstrated good to excellent reliability for both raters in all postures of the neck, shoulder, and wrist. This study demonstrated that posture assessment of manufacturing workers using Multimedia Video Task Analysis (MVTA) is a reliable method.
Valentim et al. (2018) [41]	The intra-raters’ reliability for the RULA ranged from poor to almost perfect (kappa: 0.00–0.93), and Strain Index from poor to excellent (ICC2.1: 0.05–0.99). The inter-raters’ reliability was very poor for RULA (kappa: −0.12 to 0.13) and ranged from very poor to moderate for Strain Index (ICC2.1: 0.00–0.53). The agreement was good for RULA (75–100% intra-raters, and 42.24–100% inter-raters) and to Strain Index (EPM: −1.03% to 1.97%; intra-raters, and −0.17% to 1.51% inter-raters). The internal consistency was appropriate for RULA (a = 0.88), and low for Strain Index (a = 0.65). Moderate construct validity was observed between RULA and Strain Index, in wrist/hand-wrist posture (rho: 0.61) and strength/intensity of exertion (rho: 0.39).
Stephens et al. (2006) [26]	Intraclass correlation (ICC) coefficients for task variable ratings and accompanying data ranged from 0.66 to 0.95 for both individuals and teams. The Strain Index Score ICC (2,1) for individuals and teams were 0.56 and 0.82, respectively. Intra-rater reliability for the hazard classification (tetrachoric correlation) was 0.81 for individuals and 0.88 for teams. The results indicate that the Strain Index has good test–retest reliability.
Paulsen et al. (2015) [16]	Inter-rater reliability was characterized using a single-measure, agreement-based ICC. Interrater reliability of Strain Index assessments was moderate to good (ICC = 0.59, 95% Confident Interval (CI): 0.45–0.73), a similar finding to prior studies. Inter-rater reliability of Occupational Repetitive Actions (OCRA) checklist assessments was excellent (ICC = 0.80, 95% CI: 0.70–0.89). Task complexity had a small, but non-significant, effect on inter-rater reliability Strain Index and OCRA checklist scores. Both the Strain Index and OCRA checklist assessments possess adequate inter-rater reliability for the purposes of occupational health research and practice.
Hollak et al. (2014) [39]	The ICCs were excellent (ICC > 0.91) in all proposed protocols except for the one trial Purdue Pegboard test with ICCs of 0.80–0.82. In all tests, the ICCs were higher for the two-trial protocol than for the one trial protocol. For all tests, the Limits of Agreements (LoAs) were about twice as large for the one trial protocol compared to the two-trial protocol. All two trial protocols had a variability of the LoA of lower than 16% when compared to the criterion values.
Coenen et al. (2014) [36]	Inter-observer reliability can be expressed by an ICC of 0.708 (0.511–0.873). The concurrent validity of subjective hand-arm vibration assessment in comparison to the objective measurement can be expressed by a weighted kappa of 0.535 (0.285–0.785). As a comparison, the ICC depicting the validity of the vibration values provided by the manufacturers as compared to the objectively measured vibrations was calculated 0.505 (0.364–0.706). Exact agreement of the subjective assessment compared to the objective measurement occurred in 52% of the assessed tasks. The additional analysis to investigate a possible learning effect showed 44% agreement of the subjective and objective assessment during the first two tasks of each observer while there was 59% agreement during the last two tasks.

^a^ The table may include direct quotes to maintain consistency with the original articles. Please seek the original articles for further information.

Intraclass correlation coefficients (ICCs) are broadly used in reliability analyses and are a good measure of agreement beyond chance [34]. The ICC can provide a correlation coefficient similar to that produced by a kappa statistic [46]. ICCs are derived using variance components from various analysis of variance (ANOVA) models [29]. ICCs are the most precise and comprehensive methodology and flexible given the number of ICC variations for assessing inter-rater reliability [47,48,49].

The tetrachoric correlation coefficient was designed to show the correlation between variables that have been translated from a continuous to a dichotomized value [26,50,51]. A continuous variable is translated into a categorical value for some exposure assessment tools, such as the Strain Index [21]. Specifically, the continuous variable of the Strain Index score (hazard classification score) may be translated into a dichotomous score (hazardous/not hazardous) [26]. The tetrachoric correlation coefficient would be the appropriate reliability coefficient in this case.

“A Bland–Altman plot is a useful display of the relationship between two paired variables using the same scale. It allows you to perceive a phenomenon but does not test it, that is, does not give a probability of error on a decision about the variables as would a test” [52]. Bland–Altman plots were first proposed in 1986 as an analysis based on quantifying the agreement between two measurements by analyzing the mean difference and determining the limits of agreement [53]. The analysis quantifies the bias and a range of agreement in which approximately 95% of the differences between one measurement and another can be found (roughly two standard deviations). This type of analysis does not signify what levels of agreement are acceptable. Best practice dictates that interpretations of limits of agreement be decided upon a priori using other statistically relevant data [54].

The word correlation describes a measure of a monotonic association between two or more variables and can be used to control for covariates. Monotonic refers to a relationship where when one variable increases, so does the other variable, or a relationship where one variable increases and the other variable decreases [55]. Correlation means co-relation or the degree to which two variables “go together.” Consequently, one may define linear correlation as the degree to which two random variables go together in a straight line and is often described as a Pearson Product-Moment Correlation Coefficient. “It is a numerical estimate of both the strength of the linear relationship and the direction of the relationship” [56]. This numerical estimate will be between −1 and 1, where a value of 1 indicates a positive or “perfect” dependence between the variables of interest and a value of −1 a negative or “poor” dependence. A value of zero indicates there is no linear relationship between the variables. Two conditions should be checked before using this coefficient. First, the data should come from a random and/or representative sample. Second, both variables should be continuous, jointly normally distributed, random variables that follow a bivariate normal distribution in the sample population [55].

## 4. Discussion

This review indicates that relatively few articles have investigated the reliability of observation-based ergonomics exposure assessment tools for the upper extremities. Consequently, there is a need for additional research to be completed on the reliability of ergonomics exposure assessment tools that rely on observations, including emerging tools such as the Distal Upper Extremity Tool (DUET), the revised Strain Index, and others [57,58,59,60].

Of the articles included in this review, OCRA, HAL, and the Strain Index were the most heavily researched tools. Many ergonomic risk assessment tools analyzed in studies included in this review, including but not limited to REBA and RULA, may have acceptable reliability in certain situations; however, they are susceptible to differing opinions and interpretations since the tools only analyze a “snapshot” of a work process. Analyzing a snapshot of a process does not fully indicate a process’s ergonomic risks since some moments may be considerably higher or lower risk than others. Additionally, asking the tool user to “select the most difficult task” presupposes that the tool user will know the risk scores for various “snapshots” that may be selected.

The Strain Index was one of the most repeatedly high-performing assessment tools for reliability. The study conducted by Stevens et al. [29] demonstrated that the Strain Index tool had good reliability and that the most important variable, the hazard classification, had excellent reliability. Another similar study completed by Stephens et al. [26] demonstrated similarly high reliability for the Strain Index. It is worth noting that another very high-performing method was the Multimedia Video Task Analysis (MVTA) [37]. However, it was only evaluated in one study.

The challenge of ensuring that observations remain consistent between all raters arises with any observational exposure assessment tool [40] and in test–retest studies when the studied items change over time [24,61,62]. Video recordings provide a consistent view of the process and ensure that all raters have the same information. As shown in the study by Paulsen et al. [40], a downside is that sometimes the body part to be observed is not clearly visible. Trask et al. [63] stated, “Observers rank partly visible postures on video frames differently [between raters] than fully visible postures…partly visible data, especially when observers disagree as to the level of visibility, introduces more between observer variability when compared to fully visible data”. Further, according to Dockrell et al. [38], measuring the reliability of an assessment tool while it is being used in a real-life situation may be preferable. The dissonance between the two methods (video-based analysis vs. field studies) may suggest that the best method would be to initially use video-based observations for pilot experiments, with the plan to follow up with a more robust field study to provide further statistical evidence regarding the reliability of a tool.

Given the variety of reliability coefficients, significant efforts must be taken to design how to test each exposure assessment tool. Sources of variation should be determined and controlled as much as possible. Such controls could include using video files to ensure the tasks do not change with the introduction of different workers or if the actual job is changed. Future studies should also consider the time between tests so that the study does not report the tools to be more accurate than they are, due to the raters recalling their previous ratings. Temporality may be especially important to consider in studies involving teams or methods that use a consensus approach, since the teams will have discussions which may make their conversation(s) more memorable [26,62].

Many tools have different test variables. For example, various task variables in the Strain Index combine to provide an overall risk score. The tool provides a dichotomous classification of whether the job is hazardous based on that score. In general, the reliability of each of the contributing variables of an ergonomics exposure assessment tool should be tested. Appropriate reliability coefficients should be selected for each variable. It was evident from the literature that a single reliability statistic is insufficient for a thorough reliability study. Only a combination of appropriately selected coefficients should be considered sufficient. Additionally, one should discuss the limitations of the coefficients used.

It is evident that the more raters included in a study, the stronger the study. Two studies reference the same work by Morrow & Jackson [35], suggesting that at least 30 raters should be used for reliability studies. Another aspect is that many tools try to be “user friendly” but fail to recruit a rater population representing the end-user population, which in some cases may possess less education and training than the research team. Researchers should differentiate the raters’ age and experience levels to represent the target population. Furthermore, the researchers should attempt to represent multiple industries with their analysis to support generalizability, which was listed as a limitation by many of the articles reviewed. A well-represented sample of raters may be considered a strength, such as [26]. The raters in this study comprised six graduate students and nine ergonomic practitioners (each with varying levels of experience) and were from three different cities. All raters received some level of training.

## 5. Conclusions

The results of this review indicated no singular best practice when performing rater-reliability studies. Instead, there were multiple methodological approaches researchers chose to use. Some variations in methodologies include the selection of reliability coefficients, rater and participant selection, and direct vs. digital observation. The results of this review provide professional ergonomists and other scientists a resource for assessing the reliability of available observational exposure assessment tools and information on common methods for assessing the reliability of newly developed observational risk assessment tools.

A limitation of this systematic literature review is the limited number of databases used. Including more databases may have returned more relevant articles. Similarly, selecting keywords that comprised the search strings and the decision to remove the truncation symbols and other functional symbols to allow for standardization across the platforms could have left out several relevant articles. Searching in each database separately and tailoring the search strings for each database might result in more comprehensive results.
